# Ulcerated Stenosis of the Small Intestine Associated With Hepatic Portal Venous Gas After Treatment for Cytomegalovirus Enteritis: A Case Report

**DOI:** 10.7759/cureus.59495

**Published:** 2024-05-01

**Authors:** Kimitoshi Kubo, Issei Ashida, Noriko Kimura

**Affiliations:** 1 Department of Gastroenterology, National Hospital Organization Hakodate National Hospital, Hakodate, JPN; 2 Department of Pathology, National Hospital Organization Hakodate National Hospital, Hakodate, JPN

**Keywords:** diagnosis, small intestinal stenosis, colonoscopy, portal venous gas, cytomegalovirus enteritis

## Abstract

Due to its rarity, cytomegalovirus (CMV) enteritis remains poorly described with regard to its endoscopic and radiological findings. A 75-year-old woman was admitted to our hospital with abdominal pain and was treated with an antiviral agent for CMV enteritis. She was readmitted to our hospital 10 days after discharge due to a recurrence of abdominal pain. Emergency computed tomography revealed hepatic portal venous gas (HPVG) and ileal dilatation involving focal stenosis of the ileum. The patient underwent laparoscopic partial resection of the small intestine and was finally diagnosed with ulcered stenosis of the small intestine after treatment for CMV enteritis. This report represents a valuable addition to the literature describing a rare case of ulcerated stenosis of the small intestine associated with HPVG after treatment for CMV enteritis.

## Introduction

A double-stranded DNA virus belonging to the Herpesviridae family, cytomegalovirus (CMV) has been reported to cause severe disease, particularly in immunocompromised patients due to reactivation of latent CMV infection or due to primary CMV infection [1], and the gastrointestinal tract is among the sites affected in both immunocompetent and immunocompromised patients [2], with its prevalence reported to be 12.9%, 21.3%, 8.4%, and 57.3% in the esophagus, stomach, small intestine, and colon, respectively [3].

Hepatic portal venous gas (HPVG) is a rare condition that occurs when gas produced by intestinal bacteria or present intramurally enters the portal venous circulation [4]. HPVG is reported to be caused by intestinal ischemia in a majority of cases, with its etiologies shown to include thromboembolism, vasculitis, segmental mediolytic arteriopathy, bowel obstruction, abdominal trauma, and neoplasms [4,5]. Given its mortality rate of 29-35%, surgery should be considered depending on its clinical presentation and other CT findings [6,7]. Here, we report a case of HPVG associated with ulcerated stenosis of the small intestine after treatment for CMV enteritis.

## Case presentation

**Figure 1:**
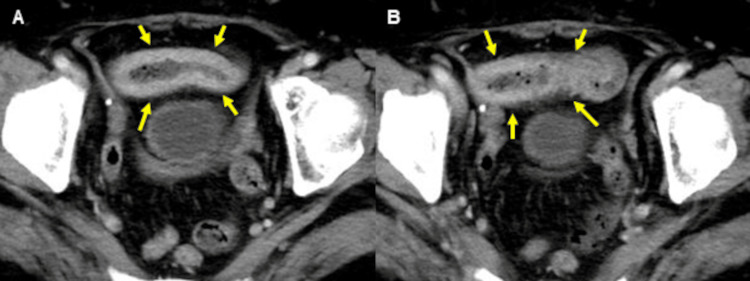
Computed tomography (CT) reveals focal wall thickening in the ileum.

**Figure 2:**
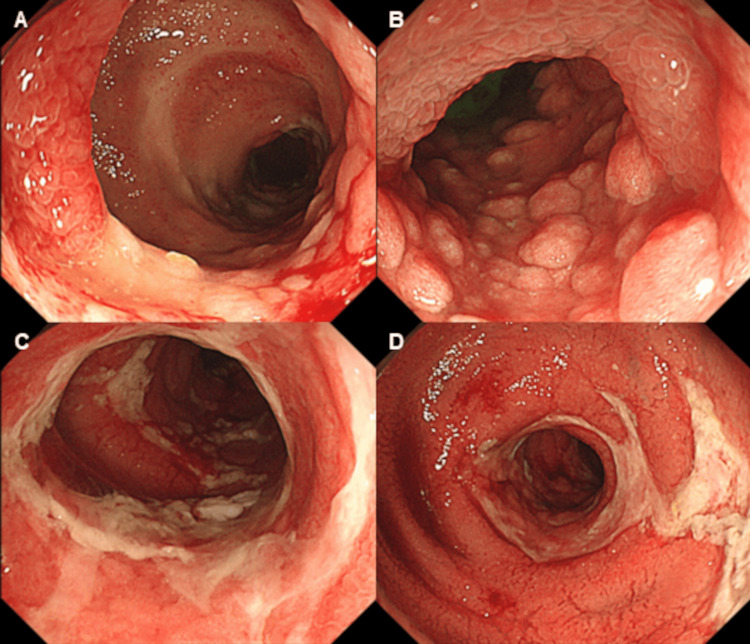
Endoscopic findings. The ileum shows a cobblestone-like ulcer (panels A and B), an annular ulcer (panel C), and a longitudinal ulcer (panel D).

**Figure 3:**
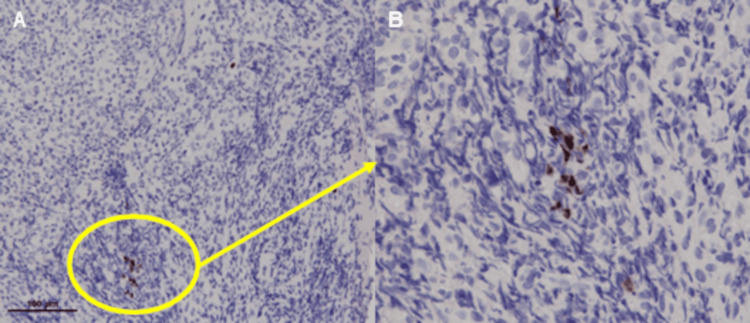
Immunohistochemistry shows the resected specimen is positive for cytomegalovirus.

**Figure 4:**
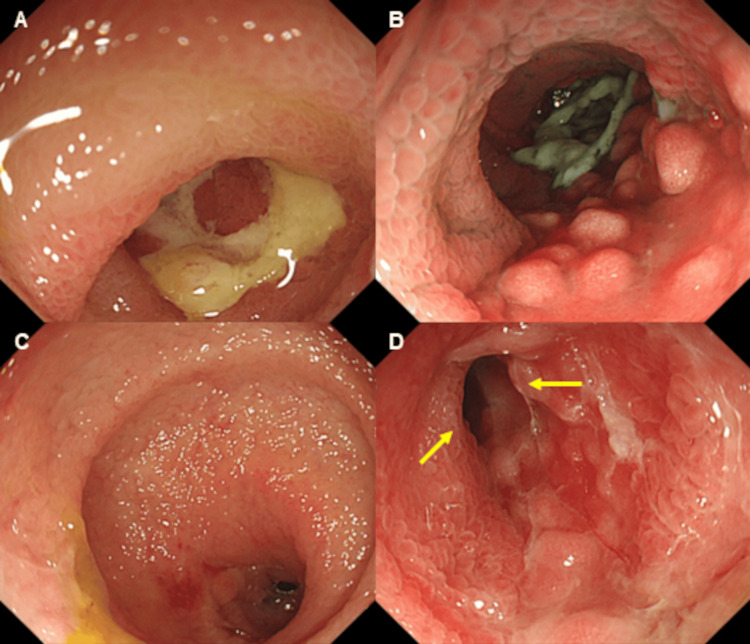
Endoscopic findings after treatment. Post-treatment colonoscopy reveals an improvement in the ulcers (panels A-C), resulting in an improvement in the stenosis of the small intestine (panel D, arrows).

A 75-year-old woman with a history of lung cancer, cerebral infarction, and hypertension was admitted to our hospital with abdominal pain and anorexia lasting for one month. Laboratory findings included: white blood cell count, 10.1 × 10^9^/L; C reactive protein, 1.2 mg/dL; serum protein, 6.2 g/dL; albumin, 3.0 g/dL; blood urea nitrogen, 12.8 mg/dL; creatinine, 0.6 mg/dL; glucose, 119 mg/dL; and glycated hemoglobin, 6.3%. Computed tomography (CT) revealed focal wall thickening in the ileum (Figure 1), while colonoscopy (CS) revealed cobblestone-like, annular, and longitudinal ulcers in the lower ileum (Figure 2). A histopathological examination of biopsy specimens, performed for differential diagnosis of CMV enteritis, Crohn’s disease, eosinophilic enteritis, and malignant lymphoma, revealed granulation tissue formation with high neutrophil infiltration. Immunohistochemistry showed the presence of CMV-positive cells (Figure 3), while the patient tested positive for anti-CMV IgG antibody, albeit being negative for CMV antigen pp65 and anti-CMV IgM antibody. She was thus diagnosed with CMV enteritis and intravenously treated with ganciclovir (5 mg/kg) for two weeks. Post-treatment CS revealed an improvement in the ulcers (Figure 4), resulting in an improvement in the stenosis of the small intestine (Figure 4D), and the patient was discharged on day 28 of hospitalization.

**Figure 5:**
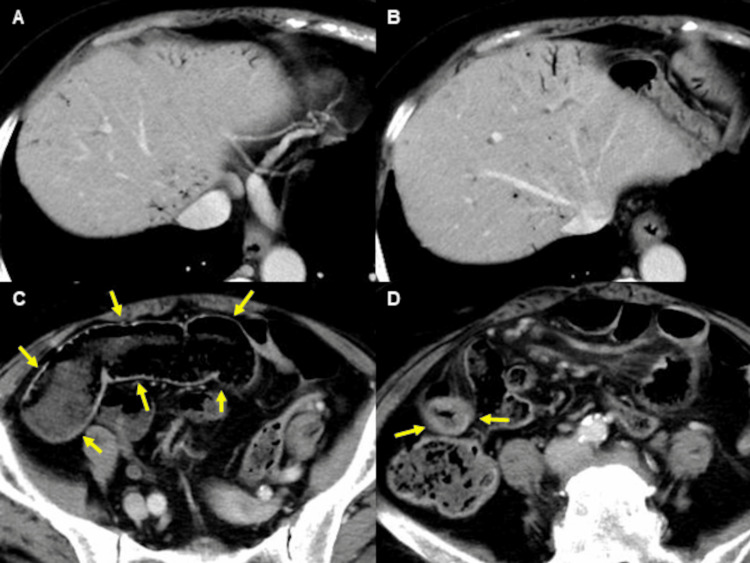
Emergency CT shows hepatic portal venous gas (HPVG) (panels A and B) and ileal dilatation (panel C, arrows) involving focal stenosis of the ileum (panel D, arrows).

However, the patient was readmitted 10 days later due to a recurrence of abdominal pain, and emergency CT revealed HPVG (Figure 5A, 5B) and ileal dilatation involving focal stenosis of the ileum (Figure 5C, 5D). Emergency diagnostic laparoscopy revealed thickening of the ileum but no findings prompting intestinal resection such as strangulation, necrosis, or perforation. She was thus treated conservatively with fasting.

**Figure 6:**
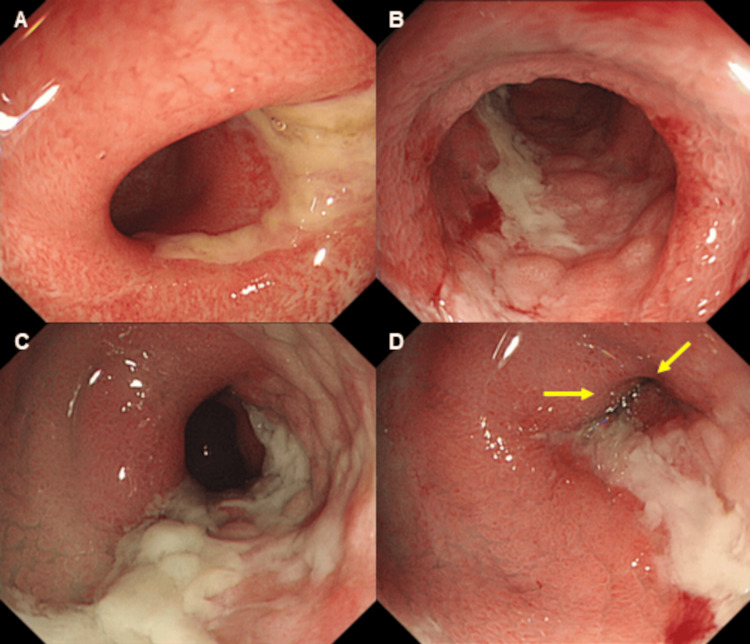
Endoscopic findings after resolution of HPVG. Colonoscopy revealed focal stenosis of the small intestine involving a longitudinal ulcer extending from its oral to anal sides (panels A-D).

CT performed seven days later showed resolution of HPVG and focal wall thickening in the ileum, while CS revealed focal stenosis of the small intestine involving a longitudinal ulcer extending from its oral to anal sides (Figure 6).

**Figure 7:**
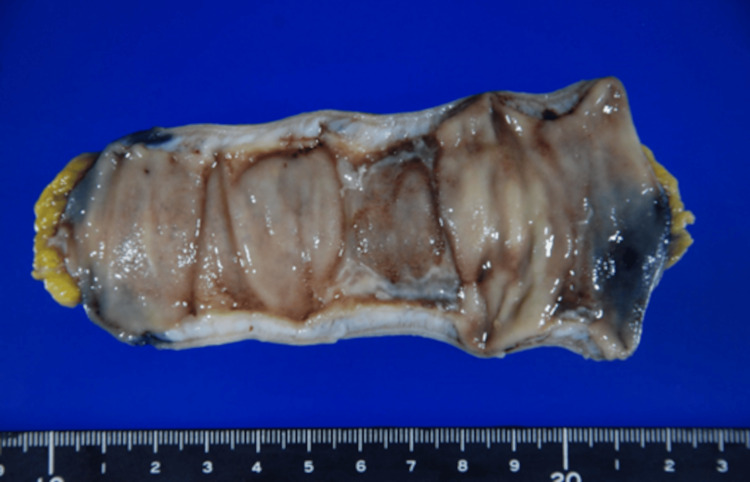
The resected specimen shows a 20 × 20 mm ulcer involving edema of the surrounding mucosa.

Laparoscopic partial resection was performed on the stenotic lesion of the small intestine after treatment for CMV enteritis, and the resected specimen revealed a 20 × 20 mm-sized ulcer involving edema of the surrounding mucosa (Figure 7), while immunohistochemistry showed no presence of CMV-positive cells.

The patient was finally diagnosed with ulcerated stenosis of the small intestine associated with HPVG after treatment for CMV enteritis, likely due to increased intestinal pressure due to stenosis and ulcer-associated mucosal damage. The patient’s course was uneventful for three years after surgery.

## Discussion

The present case highlights two important aspects of CMV enteritis.

First, CMV enteritis may lead to stenosis of the small intestine after treatment, while it is rare and has mostly been reported in case reports. Indeed, a very recent retrospective study [8] reported that of the 18 patients with CMV enteritis, 12 (66.7%) had received antiviral therapy and three (16.7%) had undergone surgery (two and one for stenosis and perforation, respectively), while their main symptoms included gastrointestinal bleeding (72.2%), abdominal pain (55.6%), and fever (33.3%), with their in-hospital and overall mortality rates shown to be 27.8% and 38.9%, respectively, and with the risk factors for CMV enteritis shown to include immunocompromised status, steroid use, shock, concurrent pneumonia, antibiotic exposure, radiotherapy, chronic kidney disease, and CMV colitis. Again, another retrospective study of patients with gastrointestinal CMV infection reported an in-hospital mortality rate as high as 23.3% among those with CMV enteritis versus 20.7% among the overall patients, with surgery being performed in as high as 20.7% of those with CMV enteritis versus 7% of the overall patients [3]. Therefore, clinicians should be aware that CMV enteritis may be complicated by perforation or stenosis, with surgery being required in a nonnegligible proportion of cases. In the present case, the patient developed abdominal pain and showed no apparent risk factors, but required surgery after treatment for CMV enteritis, due to stenosis of the small intestine associated with HPVG.

This was a case of CMV infection and associated intestinal ulcer formation, where the former was due to reactivation of latent CMV infection in an immunocompromised patient, while the latter was thought to be due to ischemic mucosal damage secondary to CMV infection of vascular endothelial cells [9,10] resulting in abnormal cellular swelling and enlargement, vascular luminal compromise, fibrin thrombus formation, local vasculitis, and damage in the intestinal tissue supplied by the affected blood vessels [10,11].

Second, CMV enteritis presents with varying endoscopic findings, which are reported to comprise three categories, i.e., ulcers, inflammation, and polypoid mass, with ulcers shown to be the most common at 72.2-76.7% [3,8] and with their morphologies reported to be irregular, longitudinal, or circumferential [3,12,13]. Consistently with these findings, our patient exhibited multiple ulcers in the lower ileum, which were cobblestone-like, annular, and longitudinal.

## Conclusions

The present case shows that treatment for CMV enteritis may lead to ulcerated stenosis of the small intestine, which, in turn, may be associated with the risk of HPVG. In addition, CMV enteritis presents with endoscopic findings as varied as irregular, longitudinal, and circumferential ulcers. Thus, clinicians need to be aware of these potential sequelae and varied endoscopic findings in the management of CMV enteritis.
